# Understanding Technology Anxiety by the Interaction between Social Support and Educational Context

**DOI:** 10.1093/geroni/igab046.3335

**Published:** 2021-12-17

**Authors:** Susanna Joo, Changmin Lee, YoonMyung Kim, Chang Oh Kim, Yun Mook Lim, Hey Jung Jun

**Affiliations:** 1 Yonsei University, Seodaemun-gu, Seoul-t'ukpyolsi, Republic of Korea; 2 Yonsei University, Seoul, Seoul-t'ukpyolsi, Republic of Korea; 3 Severance Hospital, Yonsei University College of Medicine, Seoul, Seoul-t'ukpyolsi, Republic of Korea

## Abstract

The purpose of this study was to examine the interaction effects of social support from family and educational contexts on technology anxiety among Korean older adults. We collected data by online recruiting in February 2021, and the sample was Korean older adults without dementia (N=310; 65-89 years old). The dependent variable was technology anxiety, which meant the expected degree of worry under the assumption that a wearable robot for exercise was used. Independent variables were four types of social support (emotional, instrumental, physical, and financial support) provided by family members, such as spouse, children, or siblings. The moderating variable was the binary educational context (high school and under=0; college level and over=1). Interaction effects were estimated by bootstrapping and PROCESS macro with four regression models about each type of social support. Results showed the interaction effect between physical support and educational context was significant on technology anxiety. Concretely, getting more physical support was significantly associated with a lower level of technology anxiety for highly educated older adults, while it was not significant for less-educated older adults. There was no additional type of social support which had not only significant interaction effects with educational context but also main effects on technology anxiety. It suggested that providing direct physical help, including daily care or assistance, could decrease feeling technology anxiety, especially not for less-educated seniors but for highly educated Korean older adults.

